# Acute Effects of Turmeric Extracts on Knee Joint Pain: A Pilot, Randomized Controlled Trial

**DOI:** 10.1089/jmf.2020.0074

**Published:** 2021-04-16

**Authors:** Lorena Calderón-Pérez, Elisabet Llauradó, Judit Companys, Laura Pla-Pagà, Noemí Boqué, Francesc Puiggrós, Rosa-M Valls, Anna Pedret, Josep Manuel Llabrés, Lluís Arola, Rosa Solà

**Affiliations:** ^1^ Eurecat, Technological Center of Catalonia, Nutrition and Health Unit, Reus, Spain.; ^2^ Functional Nutrition, Oxidation and Cardiovascular Diseases Research Group (NFOC-Salut), Faculty of Medicine and Health Sciences, Rovira i Virgili University, Reus, Spain.; ^3^ PLAMECA S.A. (Medicinal Plants and Food Supplements, S.A.), Barcelona, Spain.; ^4^ Nutrigenomics Research Group, Faculty of Chemistry, Rovira i Virgili University, Tarragona, Spain.; ^5^ Intern Medicine Department of Sant Joan de Reus University Hospital, Reus, Spain.

**Keywords:** knee joint pain, turmeric extracts, curcuma longa, Brewer's yeast, painkiller

## Abstract

Turmeric extracts (TEs) have been shown to be suitable as a pain treatment for human joint arthritis. In a pilot, randomized clinical trial, 68 individuals with mild/moderate knee joint pain (KJP) consumed a new formulation of water-soluble TEs and insoluble curcuminoids (B-Turmactive^®^) or brewer's yeast as a placebo for 1 week. Our hypothesis was that B-Turmactive would have a short-term analgesic effect on KJP measured by the self-reported Western Ontario and McMaster Universities Osteoarthritis Index (WOMAC). After 3 days and 1 week, both treatments reduced pain when walking on a flat surface (*P* < .01), going up or down stairs (*P* < .001), and sitting or lying (*P* < .05), but only B-Turmactive reduced pain at night while in bed and in an upright standing position (*P* < .01). Concerning global KJP, it was reduced by both treatments after 3 days and 1 week of the intervention (*P* < .001), being less with B-Turmactive after 1 week (*P* = .012 vs. 3 weeks). Although no intertreatment differences were observed, only B-Turmactive decreased high-sensitivity C-reactive protein levels (*P* = .045) at 1 week, which indicates a prompt analgesic effect mediated by a decrease in inflammatory status.

## Introduction

Pain, a major clinical problem, is poorly treated with the available therapeutics.^1^ Knee pain affects ∼25% of adults >55 years, and recently, herbal therapies with anti-inflammatory properties have begun to be developed as safe alternatives to pharmaceutical treatment.^2,3^ Turmeric extracts (TEs) from *Curcuma longa L* (turmeric) have been shown to have biological activity, including antioxidant, anti-inflammatory, anticancer, antigrowth, antiarthritic, antiatherosclerotic, antidepressant, antiaging, antidiabetic, antimicrobial, wound healing, and memory-enhancing activities.^4^

The health benefits of curcumin were recently reviewed.^5–7^ Furthermore, TEs have proven to be suitable as a pain treatment for human joint arthritis in clinical trials covering a span of 4 weeks to 4 months of treatment.^1,3^ The acute effects of TEs, however, have never been evaluated. Our hypothesis was that TEs can have a short-term analgesic effect (from 3 days to 1 week) in a similar way as has been described for a 5-day treatment with ibuprofen.^2^

B-Turmactive^®^ (constituted by Turmacin^®^ and curcuminoid Betasorb^®^) is a new formulation of dry extracts of turmeric roots (Supplementary Data S1). It is a combination of the water-soluble fraction of TEs, supposedly the main beneficial constituent of turmeric in its traditional use, with insoluble curcuminoids, with clinical anti-inflammatory effects,^4^ together with beta-cyclodextrin (E 429) in an inclusion complex. The intention of joining these two fractions of the plant is to mimic the composition of the active ingredients of the native plant as well as improve curcuminoid bioavailability. Both fractions have anti-inflammatory activity with expected different and complementary mechanisms of action. The aim of the present study was to assess the acute effects (from 3 days to 1 week) of B-Turmactive on human knee joint pain (KJP).

Sixty-eight participants, aged 18 to 65 years, were recruited from the general population at the Hospital Universitari Sant Joan (HUSJ)-Eurecat, Reus, Spain, between September 19 and December 12, 2017. Subjects were included in the study if they suffered mild/moderate-intensity KJP and scored 6–10 of 20 points according to the self-reported Western Ontario and McMaster Universities Osteoarthritis Index (WOMAC) pain subscale^8^ validated for use in the Spanish population.^9^ The exclusion criteria were as follows: (1) diagnosed active rheumatoid arthritis, other knee pathologies, pharmacological treatments, or nutraceuticals that could interfere with the condition of the study; (2) menopause with osteoporosis; (3) intolerance or sensitivity to gluten or sulfites; (4) obesity; (5) musculoskeletal disorders; and (6) history of surgery or trauma affecting the knee.

Due to the concomitant inflammatory response in subjects suffering from active arthritic conditions, they were excluded from the trial to avoid influencing baseline inflammatory markers. Informed consent was obtained from all participants before the intervention. The study was approved by the Clinical Research Ethics Committee of the Institut d'Investigació Sanitària Pere Virgili, Reus, Spain. The protocol and trial were in accordance with the Helsinki Declaration and Good Clinical Practice guidelines of the International Conference of Harmonization and Good Clinical Practice (ICH GCP). The trial was prospectively registered at ClinicalTrials.gov: number: NCT03202901. This trial was reported in accordance with the CONSORT 2010 Statement (Supplementary Table S3).

A pilot, randomized, parallel, double-blind, placebo-controlled clinical trial was conducted with B-Turmactive (500 mg of TEs combined with 19.5 mg of curcuminoid complex) or brewer's yeast (BY) (200 mg of heat-inactivated yeast together with 164 mg of excipients) as a placebo. The active constituents were 10–15% turmerosaccharides and 17–21% total curcuminoids (Supplementary Data S2). The qualitative testing procedures are described in Supplementary Data S3. Participants ingested 1 capsule/day for 1 week. The randomized allocation sequence to the treatment or placebo group was at a ratio of 1:1 and it was generated with G*Power 3.1 (© 2010–2018 Heinrich-Heine-Universität Düsseldorf)^10^ by an independent researcher with no clinical involvement in the trial. Blinding was maintained by using identical opaque envelopes for B-Turmactive and the placebo. They were dispensed in opaque sealed envelopes and sequentially numbered by codes 111 and 222 according to the randomization schedule. Both participants and researchers were blinded to the allocation until the end of the intervention.

A detailed medical history was performed at baseline by a trained physician to identify KJP symptoms. A list of restricted foods, as well as products and supplements rich in curcuma, hyaluronic acid, magnesium, and silicon, was provided to both groups at baseline and monitored by using adapted food consumption questionnaires at baseline and at 1 week. Participants were also asked to maintain their habitual physical activity, which was monitored through the Physical Activity Questionnaire Class AF.^11^ Fasting blood samples were obtained at baseline and at 1 week. Samples were stored at −80°C in the central laboratory's Biobanc-REUS-IISPV until required for batch analyses.

All participants reported KJP, at least for several months. The intensity of pain was assessed through the five items for pain from the WOMAC pain subscale, administered at an in-person visit at baseline and at 1 week, and by a phone call at day 3. Each participant was asked to rate their usual pain intensity on average over the last week. In addition to the WOMAC subscale, we also measured key inflammation markers, such as the high-sensitivity C-reactive protein (hsCRP), which can be considered an objective marker of pain.^12^ Anthropometric data (body–mass index and waist circumference) were obtained with participants wearing lightweight clothing and no shoes. Biochemical analyses were performed using standardized, enzymatic automated methods in an autoanalyzer (Beckman Coulter-Synchron, Galway, Ireland); hsCRP was determined by automated immunoturbidimetry (Roche Diagnostics Systems, Madrid, Spain), and interleukin (IL) 1*β* and IL-6 were determined by ELISA kits (Abcam, Cambridge, UK).

Sample size was estimated assuming a type I error of 0.05 (two-sided) and at least 80% power for detecting a reduction of 2.6 U in the WOMAC Global Pain Scale between groups. The standard deviation was estimated to be 3.6 U.^11^ A dropout rate of 10% was estimated. Based on this, a sample size of 68 individuals was required. The normality of variables was assessed with the Kolmogorov–Smirnov test. Nonparametric variables were log transformed when possible. We used the Kruskal–Wallis test or one-factor analysis of variance to determine differences in baseline characteristics. Analyses were performed on an intention-to-treat basis. Intratreatment comparisons were assessed by a general linear model with age and sex as covariates or by the Wilcoxon test. Comparisons between treatments were carried out with an analysis of covariance model adjusted for age and sex or with the Mann–Whitney test. Statistical significance was defined as a *P* value <.05 for a two-sided test. We performed analyses by using SPSS for Windows, version 21 (IBM Corp., Armonk, NY, USA).

**FIG. 1.**
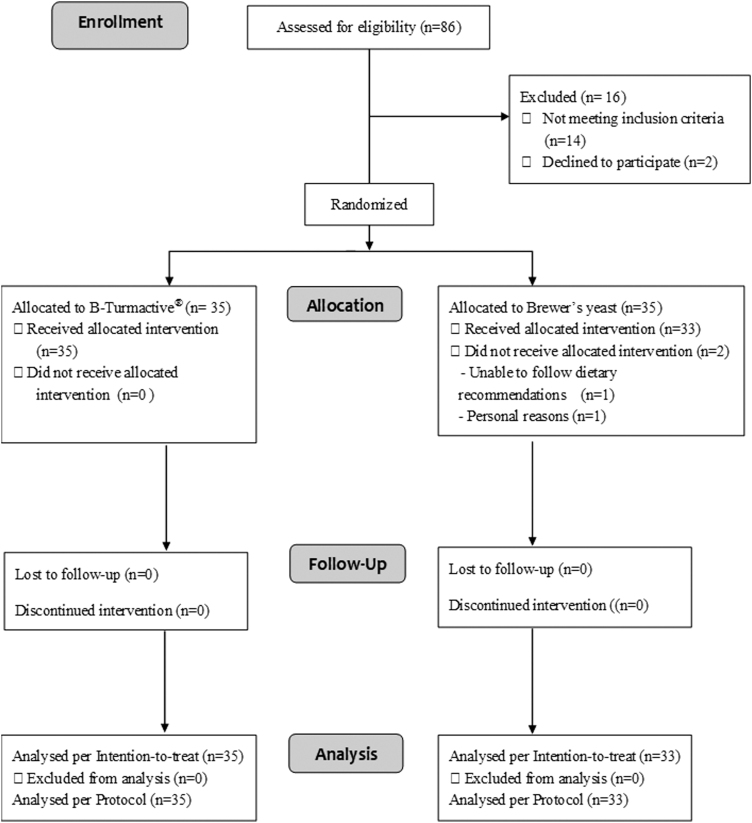
B-Turmactive^®^ CONSORT 2010 flow diagram.

Of 86 subjects who were assessed for eligibility, 14 were excluded for not meeting the inclusion criteria for KJP and 2 declined to participate. The remaining participants were allocated to the B-Turmactive (*n* = 35) and BY (*n* = 33) groups. The CONSORT 2010 flow diagram is shown in Figure 1.

**FIG. 2.**
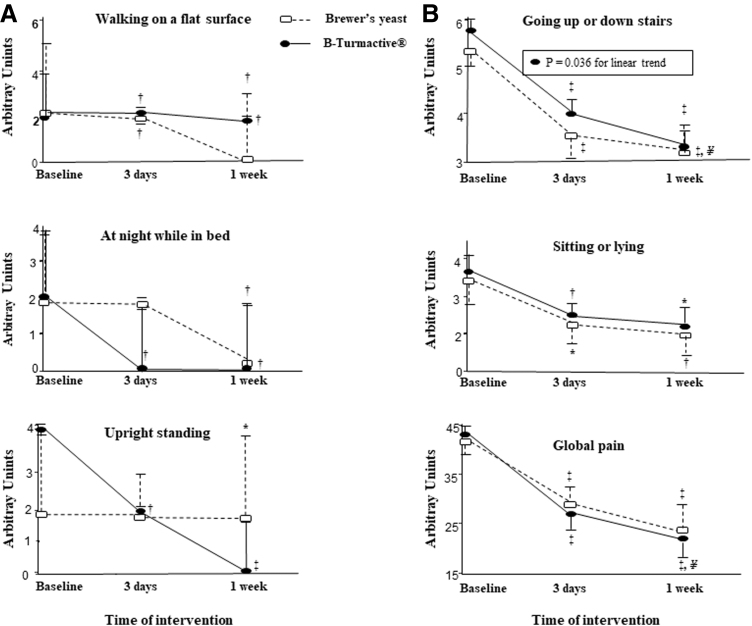
Intensity of pain, assessed using the WOMAC subscale, at baseline and after 3 days and 1 week of the intervention. Global pain was assessed as the sum of the 5 subitems of the pain subscale from the WOMAC. Panel A, nonparametric variables expressed as median (75th percentile). Panel B, parametric variables expressed as mean (standard error). *P < .05; ^†^*P* < .01; and ^‡^*P* < .001 versus baseline. ^¥^*P* < .05 versus 3 days. WOMAC, Western Ontario and McMaster Universities Osteoarthritis Index.

A total of 29 males and 39 females were enrolled in the study. No differences in baseline characteristics were observed among the intervention groups (Supplementary Table S1). Physical activity was similar among groups. No adverse effects were observed. The intensity of pain throughout the study is depicted in Figure 2.

At day 3, both placebo and B-Turmactive reduced pain when walking on a flat surface (*P* = .002 and *P* = .003, respectively), going up or down stairs (*P* < .001 for both treatments), and sitting or lying (*P* = .002 and *P* = .044, respectively), but only B-Turmactive reduced pain at night while in bed (*P* = .005) and in an upright standing position (*P* = .001). After 1 week, both the placebo and B-Turmactive reduced all types of pain referred to before: walking on a flat surface (*P* = .004 and *P* = .002, respectively), going up or down stairs (*P* < .001 for both treatments), and sitting or lying (*P* = .013 and *P* = .014, respectively), and B-Turmactive showed a greater reduction when going up or down stairs (*P* = .045 vs. 3 weeks, *P* = .036 for the linear trend). Concerning global pain (sum of all types), it was reduced by both treatments at day 3 and at 1 week (*P* < .001 for all), being lower after B-Turmactive treatment at 1 week (*P* = .012 vs. 3 weeks). No intertreatment differences were observed.

No changes in anthropometric measurements or routine biochemical parameters were observed (Supplementary Table S2). Of the inflammatory markers, only hsCRP decreased significantly after B-Turmactive treatment compared with the placebo (*P* = .048) (Table 1).

**Table 1.** Changes in Inflammatory Markers One Week After the Intervention

Variable	Intervention				Comparison of changes	
	Placebo (*n* = 33)		B-TURMACTIVE^®^ (*n* = 35)		B-TURMACTIVE vs. placebo	
	Baseline (mean ± SD)	One week (mean ± SD)	Baseline (mean ± SD)	One week (mean ± SD)	Mean (95% CI)	P
hsCRP, *mg/dL (log)*	−0.99 ± 0.36	−0.96 ± 0.36	−0.93 ± 0.40	−1.00 ± 0.41^*^	−0.104 (−0.21 to −0.001)	.048
IL-1*β*, *pg/mL (log)*	1.40 ± 0.25	1.48 ± 0.22^**^	1.34 ± 0.3024	1.39 ± 0.24	−0.035 (−0.15 to 0.08)	.532
IL-6, *pg/mL (log)*	0.008 ± 0.23	0.056 ± 0.34	0.004 ± 0.36	0.010 ± 0.35	−0.001 (−0.13 to 013)	.994

^*^ *P* = .045 and ^**^P = .010 versus baseline.

Variables log transformed for reaching parametricity. ANCOVA model adjusted by sex and age.

95% CI, confidence interval at 95%; ANCOVA, analysis of covariance; hsCRP, high-sensitivity C-reactive protein; IL, interleukin; SD, standard deviation.

This pilot study has demonstrated the short-term efficacy of B-Turmactive, a formulation of TEs combined with insoluble curcuminoids, for self-reported KJP. Our results agree with those of a recent meta-analysis of randomized clinical trials, with a longer time span of treatments, which suggested that oral administration of curcumin reduced arthritis symptoms when measured by the WOMAC scale.^3^ From our results, both BY and B-Turmactive acted as very short-term painkillers for joint pain, with B-Turmactive encompassing a broader spectrum for all types of pain and the quickest response. The analgesic effect of B-Turmactive seems to be progressive, with values at 1 week being lower than those at 3 days, and is supported by a concomitant decrease in hsCRP, a key marker of inflammation. These results agree with those of previous studies reporting anti-inflammatory effects of turmeric through modulation of transcription factors and proinflammatory cytokines.^1,13^

The daily doses of curcuminoids administered in our study were below those described for improving pain, less than 2000 mg/day, without any noticeable adverse effects.^3^ Although several human studies have reported low systemic bioavailability of curcumin after short-term oral administration (1 to 4 h) of a single dose,^3,14^ the B-Turmactive^®^ formulation combined water-soluble TEs and insoluble curcuminoids with beta-cyclodextrin as a complexation agent to increase aqueous solubility and the corresponding bioavailability.^15,16^

Randomized, placebo-controlled clinical trials are those required to prove the efficacy of a treatment. However, such proof is difficult in the presence of large placebo effects, as seen in the setting of symptomatic treatments for knee osteoarthritis.^17^ BY was used like a placebo based on the idea that the heat-inactivated *Saccharomyces* present in the formulation has no anti-inflammatory effects, unlike the active forms.^18^ In addition, the 200 mg/day of yeast used in the present study was lower than 500 mg/day of yeast, which has been shown to improve metabolic risk factors for diabetes.^19^ Despite this, the fact that BY reduced some of the types of pain was unexpected and indicates bioactivity also for the heat-inactivated form.

In this sense, our data are the first to report an acute analgesic effect (global pain reduction of 5.0 points at 1 week) of heat-inactivated forms of *Saccharomyces* on human KJP. Nonetheless, the placebo selection and the short duration of the study could be considered limitations, although this time range permitted us to support our hypothesis that TEs reduce self-reported pain on a short-term basis. Additionally, there is an inability to assess potential interactions between capsules and other diet components, and the results are limited to individuals with mild/moderate KJP. As a strength, treatments were administered alone, thus avoiding possible confusion with other components with more elaborated matrices.

In conclusion, B-Turmactive and BY reduced self-reported, mild/moderate joint pain after 3 days and 1 week of treatment when KJP was assessed as global pain. Pain at night while in bed or in an upright standing position at 3 days, however, was only reduced by B-Turmactive. The analgesic effect of B-Turmactive was progressive and accompanied by an objective decrease in inflammatory status, which suggests that it could be safely used as an emergency painkiller for KJP. To the best of our knowledge, this is the first study showing that B-Turmactive and BY improved subjective symptoms of KJP after short-term use.

## Supplementary Material

Supplemental data

Supplemental data

Supplemental data
