# Supplementary material for: Acute Effects of Turmeric Extracts on Knee Joint Pain: A Pilot, Randomized Controlled Trial
Source: J Med Food. 2021 Apr 16;24(4):436–40. doi: 10.1089/jmf.2020.0074 (PMC8080919; doi:10.1089/jmf.2020.0074)
Supplement: Supplemental data [file Supp_Data.pdf]

Supplementary Data

SUPPLEMENTARY DATA S1. TYPE AND CONCENTRATION OF EXTRACTION SOLVENT AND RATIO OF DRUG: EXTRACT

Turmacin®: solvent: water 100%. Extraction ratio 12–14:1. Excipients: silicon dioxide (0–2%).

Curcuminoid Betasorb®: solvents: methanol, acetone, and water. Three steps of extraction are performed, and each step uses one of the mentioned solvents at 100% concentration. The extraction ratio was 4–5:1. Excipient: beta cyclodextrin (E 459) 19–83%.

Raw materials are tested as per specification. This testing involves identification tests. The tests are performed by the Quality Control (QC) Department of Natural Remedies, and the QC Head is responsible for the release of raw materials. A retention sample of raw materials is stored by the QC Department. The reference number is the same as that of the batch number assigned for the material. One sample for the botanical reference material is available in the QC Department. The sample is authenticated by NISCAIR, Government of India, and the voucher specimen is deposited there.

SUPPLEMENTARY DATA S2. COMPOSITION OF PRODUCTS (INGREDIENTS PER CAPSULE)

B-Turmactive® composition

Turmacin® powder, 500 mg	*Curcuminoid Betasorb®, 19.5 mg*	*Other excipients*
Turmeric polysaccharides: 12.5%<br>Total ash content: 31.08%<br>Polyphenols: 11.46%<br>Flavonoids: 2.76%<br>Dextrins: 11.02%<br>Saponins: 9.63%<br>Protein: 6.05%<br>Terpenoids: 4.53%<br>Moisture: 3.94%<br>Bitter principles: 2.2%<br>Crude fat: 1.99%<br>Alkaloids: 0.12%	β-Cyclodextrin (E-459): 15.8 mg<br>Curcuminoids: 3.7 mg	Vitamin C (ascorbic acid): 22.5 mg<br>Diglyceride fatty acids (E-471): 20 mg<br>Magnesium stearate (E-470b): 5 mg<br>Silicon dioxide (E-551): 5 mg

Placebo composition:

Brewer's yeast, 200 mg	*Other excipients*
Inactive yeast: 98.5%<br>Inactivated yeast species: *Saccharomyces cerevisiae*	Microcrystalline cellulose (E-460): 120 mg<br>Compritol® E ATO (E-471): 40 mg<br>Magnesium stearate (E-572): 4 mg

SUPPLEMENTARY DATA S3. QUALITATIVE TESTING

Product's chemical fingerprint: in-house method. The tests are performed by the QC Department of Natural Remedies and the QC Head is responsible for the release of products. Product retention samples are stored by the QC Department. The reference number is the same as that of the batch number assigned for the material.

(Standardization: turmerosaccharides (bioactive polysaccharides are contributors to the biological effects of Turmacin®), measured by HPLC. Total curcuminoids (curcumin, demethoxycurcumin, and bisdemethoxycurcumin) measured by HPLC.

Supplementary Table S1. Basal Characteristics of Participants According to the Intervention Group

Variable	*Placebo (n=35)*	*B-TURMACTIVE® (n=35)*	P
Age, years	49.3 ± 12.7	48.1 ± 13.8	.727
Female, %	51.4	62.9	.337
Smoking habits, %			
Never	65.7	51.4	.324
Smoker	2.9	14.3	.217
Ex-smoker	31.4	34.3	.367
SBP, mm Hg	126 ± 15.	120 ± 16.3	.101
DBP, mm Hg	77 ± 10.2	74 ± 10.6	.143
Weight, kg	72 ± 12.8	69 ± 10.3	.308
BMI, kg/m^2	25.4 ± 3.6	24.7 ± 2.8	.362
Waist circumference, cm	89.0 ± 11.6	86.2 ± 9.2	.259
Waist/height	0.53 ± 0.07	0.51 ± 0.06	.439
Conicity index	1.25 ± 0.10	1.23 ± 0.09	.337
Physical activity, AU (log)	0.47 ± 0.19	0.48 ± 0.17	.912

Data expressed as mean ± standard deviation or percentage.

AU: 0, inactive; 1, very low activity; 2, low activity; 3, moderate activity; and 4, high activity. P for ANOVA or χ^2.

ANOVA, analysis of variance; mAU, arbitrary units; BMI, body–mass index; DBP, diastolic blood pressure; SBP, systolic blood pressure.
