# Supplementary material for: Acute Effects of Turmeric Extracts on Knee Joint Pain: A Pilot, Randomized Controlled Trial
Source: J Med Food. 2021 Apr 16;24(4):436–40. doi: 10.1089/jmf.2020.0074 (PMC8080919; doi:10.1089/jmf.2020.0074)
Supplement: Supplemental data [file Supp_Table2.pdf]

Supplementary Table S2. Changes in Anthropometric and Routine Biochemical Parameters One Week After the Intervention

	Intervention				*Comparison of changes*	
	Placebo (n=*33)*		*B-TURMACTIVE®* (n=*35)*		*B-TURMACTIVE® vs. placebo*	
	Post-int (Mean ± SD)	*Change Mean (95% CI)*	*Post-int (Mean ± SD)*	*Change Mean (95% CI)*	*Mean (95% CI)*	P
Weight, kg	72.6 ± 13.4	–0.117 (–0.31 to 0.08)	69.5 ± 10.2	–0.082 (–0.27 to 0.11)	0.035 (–0.24 to 0.31)	.803
BMI, kg/m^2	25.3 ± 3.69	–0.042 (–0.11 to 0.03)	24.7 ± 2.87	–0.029 (–0.09 to 0.04)	0.013 (–0.08 to 0.11)	.793
Waist circumference, cm	88.4 ± 12.2	–0.014 (–0.46 to 0.49)	86.6 ± 10.2	0.275 (–0.20 to 0.76)	0.261 (–0.42 to 0.94)	.443
Waist/height	0.52 ± 0.07	0.000 (–0.003 to 0.003)	0.52 ± 0.06	0.002 (–0.001 to 0.005)	0.002 (–0.002 to 0.006)	.437
Conicity index	1.24 ± 0.09	–0.001 (–0.006 to 0.008)	1.23 ± 0.09	0.005 (–0.02 to 0.01)	0.004 (– 0.006 to 0.01)	.412
Physical activity, AU (log)	0.52 ± 0.10	0.053 (–0.00 to 0.11)	0.52 ± 0.08	0.043 (–0.00 to 0.01)	–0.010 (–0.08 to 0.06)	.786
Glucose, mg/dL	86.4 ± 8.68	0.136 (–2.2 to 2.5)	83.6 ± 6.82	1.186 (–1.1 to 3.4)	1.051 (–2.2 to 4.3)	.524
Total Cholesterol, mg/dL	192 ± 27.5	2.23 (–2.9 to 7.4)	186 ± 35.8	–4.10 (–9.1 to 0.93)	–6.33 (–13.6 to 0.93)	.086
Triglycerides, mg/dL	86.7 ± 34.6	0.706 (–7.9 to 9.3)	85.6 ± 38.5	–6.04 (–14.4 to 2.3)	–6.74 (–18.8 to 5.3)	.270
Alanine transaminase, UI/L	15.4 ± 5.34	–0.555 (–2.0 to 0.90)	18.1 ± 9.13	–1.16 (–2.6 to 0.25)	–0.607 (–2.6 to 1.4)	.553
Aspartate transaminase, UI/L	17.9 ± 4.19	0.142 (–1.1 to 1.4)	19.7 ± 6.34	0.066 (–1.2 to 1.3)	–0.076 (–1.8 to 1.7)	.931

ANCOVA model adjusted by sex and age.

Post-int, postintervention; 95% CI, confidence interval at 95%; ANCOVA, analysis of covariance.
